# Types of nasal deformities in individuals seeking rhinoplasty at governmental hospital in Saudi Arabia

**DOI:** 10.3389/fsurg.2024.1426170

**Published:** 2024-09-24

**Authors:** Abdullah D. Alotaibi, Bashayr N. Alsuwayt, Rana N. Raghib, Rahaf S. Alsayer, Sarah M. Albarrak, Abdullah N. Alrasheedi, Mohd Saleem, Sheeba Afreen, Oren Friedman

**Affiliations:** ^1^ Department of Otolaryngology, College of Medicine, University of Hail, Hail, Saudi Arabia; ^2^ College of Medicine, University of Hail, Hail, Saudi Arabia; ^3^ Department of Otolaryngology, College of Medicine, Jouf University, Jouf, Saudi Arabia; ^4^ Department of Microbiology, College of Medicine, University of Hail, Hail, Saudi Arabia; ^5^ Department of Personalized and Molecular Medicine, Era Medical University, Lucknow, India; ^6^ Department of Otolaryngology, University of Pennsylvania, Philadelphia, PA, United States

**Keywords:** nasal deformities, rhinoplasty, external nasal deviation, dorsal hump deformity, nasal tip

## Abstract

**Introduction:**

This study explores the profound impact of nasal structure on individuals' self-image and emotional well-being, emphasizing the increasing popularity of rhinoplasty in Saudi Arabia, influenced by societal beauty standards portrayed on social media. The investigation aims to unravel the complex interplay between demographic factors, such as gender and age distribution, and prevalent nasal deformities in a cohort of 293 participants.

**Material and methods:**

This retrospective study at the University of Hail and King Khalid Hospital, Saudi Arabia, investigated nasal deformities in 293 participants aged 15–54. Ethical approval was obtained, and data, including bio-demographics and nasal deformities, were retrospectively reviewed. Statistical analyses, utilizing chi-square and Fisher exact tests, assessed associations, enhancing internal validity. The study targeted a diverse population, emphasizing ethical guidelines and systematic sampling.

**Results:**

Our study of 293 participants revealed a prevalence of common nasal deformities. Dorsal hump deformity (59.0%) was the most prevalent, followed by external nasal deviation (54.6%). Significant gender differences were observed, with males more prone to external nasal deviation (65.6%), while decreased nasal tip rotation was more common in females (40.6%). Variations in nasal tip shape were statistically significant, with broad nasal tip shape more prevalent in females (35.2%).

**Conclusion:**

In conclusion, our study highlights the prevalence of common nasal deformities, emphasizing significant gender variations. These findings contribute to a deeper understanding of nasal anatomy, essential for informed decision-making in rhinoplasty.

## 1 Introduction

The facial prominence of the nose makes it a significant aesthetic focal point, contributing significantly to an individual's self-image, self-confidence, and overall sense of self-worth. Disruptions in nasal structure, stemming from factors like surgery, trauma, or natural variations, can have profound emotional implications for the person (1). Individuals' discontent with their nose's appearance may lead them to choose to undergo rhinoplasty, a cosmetic surgical procedure designed to modify the nose's shape or aesthetics while maintaining or improving nasal airflow (2). Facial cosmetic procedures like rhinoplasty and revision rhinoplasty have significant impacts on both the aesthetic appearance and psychological well-being of individuals (1). In Saudi Arabia, there has been a notable rise in the frequency of rhinoplasty procedures, which constitute 30% of all cosmetic surgeries, in recent years. This surge is attributed not only to the motivation to address dysmorphic facial features but also to the increasing influence of societal beauty standards portrayed on social media, which plays a substantial role in shaping individuals' choices (3). The decision to undergo a cosmetic procedure is significantly impacted by prolonged exposure to cosmetic surgery-related content on social media, coupled with the portrayal of features deemed ideal (4). Reduction rhinoplasty, in particular, carries inherent risks. While severe complications are infrequent, numerous short- and long-term issues may arise, potentially resulting in dissatisfaction with the aesthetic outcome, disappointment for the patient, and even functional issues (5). Complications associated with rhinoplasty can be categorized into two groups: aesthetic issues, which may necessitate revision rhinoplasty, and non-aesthetic concerns (6), including breathing difficulties reported by 70% of revision rhinoplasty patients. Additionally, harm to the skin and soft tissues can lead to atrophy, fibrosis, numbness, and the development of cysts resulting from displaced mucosa or subcutaneous granulomas. Although infections are uncommon, their occurrence can be life-threatening, especially when sinus surgery and rhinoplasty are performed together (7). Recognizing the potential complications is a crucial aspect of an individual's decision-making process when considering rhinoplasty. The aim of this study is to investigate the intricate relationship between demographic factors, including gender and age distribution, and prevalent nasal deformities among a sample of 293 participants.

## 2 Materials and methods

### 2.1 Study design and setting

This study employed a retrospective design to comprehensively investigate the prevalence of common nasal deformities in a diverse participant population. The study was conducted at the University of Hail and King Khalid Hospital, situated in Saudi Arabia. Ethical approval for the study was obtained from the Institutional Review Board (IRB) of the University of Hail. This step ensured that the research adhered to ethical guidelines and safeguarded the rights and well-being of the participants. The IRB's approval underscored the ethical conduct and scientific validity of the study.

### 2.2 Population and sampling

Data collection involved a retrospective chart review of the medical records of patients seeking rhinoplasty. The information extracted included bio-demographic details such as gender, age, and educational qualifications, as well as the presence or absence of specific nasal deformities. The systematic data collection process ensured the accuracy and completeness of the information gathered. The study population consisted of individuals aged 15–55 years. This age range was selected to capture a broad spectrum of participants while excluding those below 15 years and ensuring relevance to individuals seeking rhinoplasty. A total of 293 participants were included in the study. All patients were operated by senior author through open approach starting with inverted v incision, elevating the nasal skin flap identifying the dorsal hump and removing it followed by using spreader flaps or grafts especially if the nasal hump was big or the upper lateral cartilages were weak. For the nasal tip reconstruction, if the projection was good then columellar strut used to support it. If the tip was ptotic or under projected, then septal extension graft used. If more tip projection needed, then different tip grafts were used. If the septal cartilage was insufficient, auricular cartilage or rib cartilage used however, this scenario was in minority of cases since all cases were primary. The indication for surgery includes improvement of the appearance of nose with or without improvement of anatomic nasal air way obstruction.

### 2.3 Inclusion and exclusion criteria

In this study, the inclusion criteria were carefully defined to ensure the relevance and completeness of the participant cohort. Participants aged between 15 and 54 years were included, aligning with the demographic of individuals commonly seeking rhinoplasty. The chosen age range aimed to provide a comprehensive understanding of nasal deformities in the context of individuals considering cosmetic nasal procedures. Additionally, participants were required to have complete and available data within the specified medical records, guaranteeing the accuracy and thoroughness of the information collected.

Conversely, the exclusion criteria were established to maintain the integrity and specificity of the study. Individuals below the age of 15 were excluded from the analysis, as the focus was on adults seeking rhinoplasty. Furthermore, participants with a history of previous nasal surgeries were excluded to minimize confounding variables and ensure a more homogenous representation of individuals with primary nasal deformities. These stringent criteria were implemented to enhance the internal validity of the study and facilitate a more targeted exploration of nasal deformities in the specified population.

### 2.4 Data analysis

Statistical analyses were conducted using IBM SPSS software, version 21. The choice of statistical analysis tools was based on the nature of the data and the research questions posed. Chi-square and Fisher exact tests were utilized for assessing associations between bio-demographic variables and nasal deformities, with odds ratios and 95% confidence intervals calculated to quantify the strength of these associations.

## 3 Results

### 3.1 Distribution of cases according to bio-demographic profile

**Figure 1:**
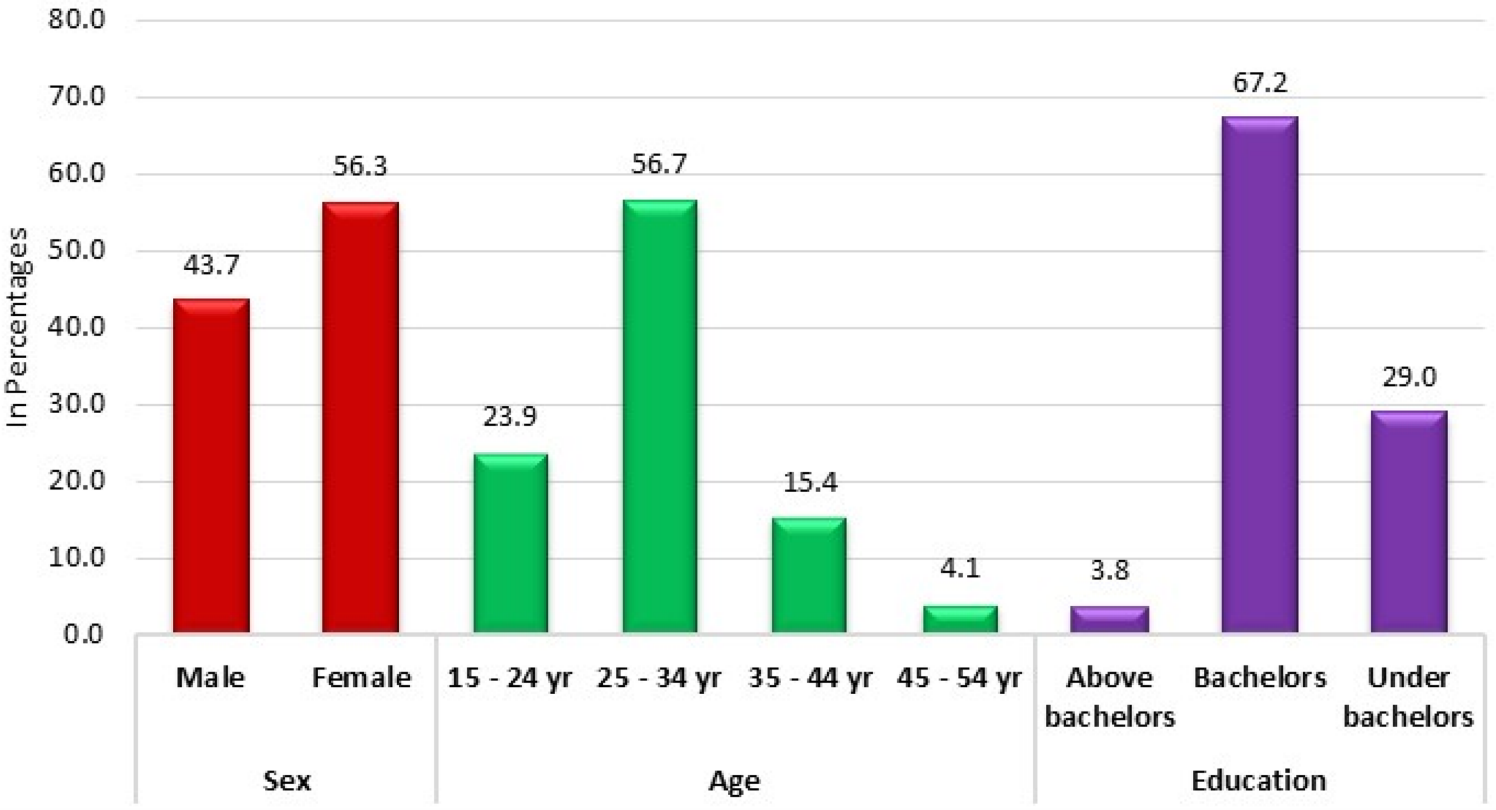
Distribution of cases according to bio-demographic profile.

In a comprehensive study encompassing 293 participants, the demographic composition was analyzed to provide insights into the sample's bio-demographic distribution. Regarding gender distribution, the cohort was fairly balanced but slightly skewed towards females, constituting 56.3% (*n* = 165) of the total participants. In contrast, males accounted for 43.7% (*n* = 128) of the sample. Age-wise segmentation of the participants revealed a predominant representation in the younger age brackets. Specifically, individuals aged between 25 and 34 years constituted the largest group, making up 56.7% (*n* = 166) of the cohort. The 15–24-year-old age group followed, representing 23.9% (*n* = 70) of the participants. Meanwhile, the older age categories, namely 35–44 years and 45–54 years, were less represented, accounting for 15.4% (*n* = 45) and 4.1% (*n* = 12), respectively. When considering educational qualifications, a majority of the participants held bachelor's degrees, comprising 67.2% (*n* = 197) of the sample. Those with educational attainment below the bachelor's level were also notable, constituting 29.0% (*n* = 85) of the participants. In contrast, a smaller fraction, 3.8% (*n* = 11), possessed qualifications above a bachelor's degree (Table 1; Figure 1).

**Table 1:** Distribution of cases according to Bio-demographic profile.

Bio-demographic variable		No. (*N* = 293)	%
Sex	Male	128	43.7
	Female	165	56.3
Age	15–24 years	70	23.9
	25–34 years	166	56.7
	35–44 years	45	15.4
	45–54 years	12	4.1
Education	Above bachelors	11	3.8
	Bachelors	197	67.2
	Under bachelors	85	29.0

### 3.2 Prevalence of common nasal deformities

**Figure 2:**
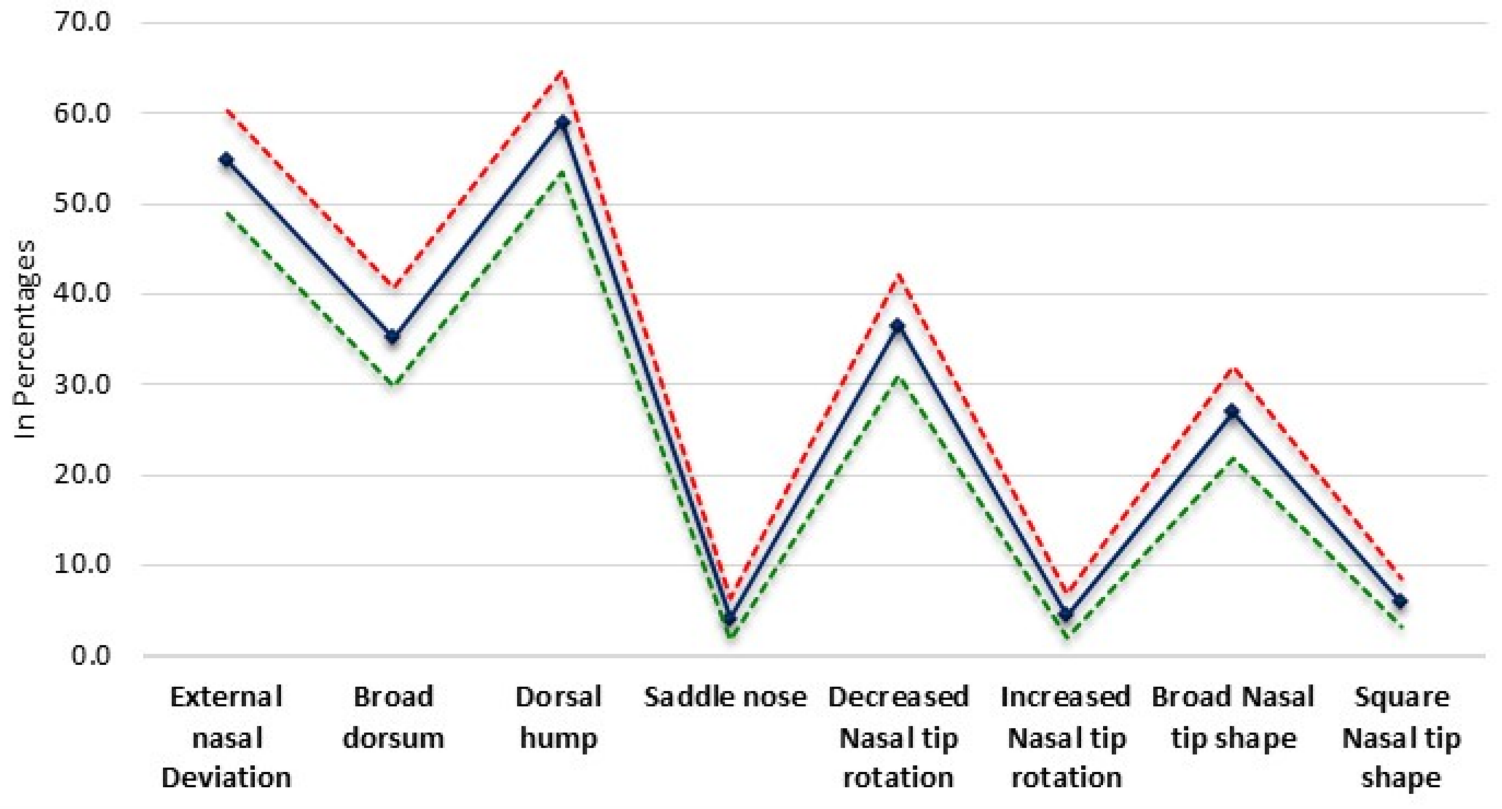
Prevalence of common nasal deformities.

The study found that the dorsal hump was the most prevalent deformity affecting 59.0% (*n* = 173) of the participants, accompanied by a CI of 53.41% –64.68%. Following closely, external nasal deviation was noted in 54.6% (*n* = 160) of the individuals, with a 95% confidence interval (CI) ranging from 48.91% to 60.31%. Broad dorsum, another notable nasal trait, was observed in 35.2% (*n* = 103) of the participants, with its CI spanning from 29.69% to 40.62%. Among the less common deformities, the saddle nose deformity was identified in a mere 4.1% (*n* = 12) of the sample, with a CI of 1.83%–6.36%. Similarly, increased nasal tip rotation was a rare occurrence, noted in 4.4% (*n* = 13) of the cohort and having a CI of 2.08%–6.79%. In contrast, decreased nasal tip rotation was more prevalent, affecting 36.5% (*n* = 107) of the participants, with a CI of 31.01%–42.03%. Lastly, variations in nasal tip shape were also observed. A broad nasal tip shape was found in 27.0% (*n* = 79) of the individuals, with a CI of 21.88%–32.04%. Conversely, a square nasal tip shape was less frequently noted, affecting 5.8% (*n* = 17) of the sample and having a CI of 3.13%–8.48% (Table 2; Figure 2).

**Table 2:** Prevalence of common nasal deformities.

Nasal deformity	No. (*N* = 293)	%	95% CI
External nasal deviation	160	54.6	(48.91–60.31)%
Broad dorsum	103	35.2	(29.69–40.62)%
Dorsal hump	173	59.0	(53.41–64.68)%
Saddle nose	12	4.1	(1.83–6.36)%
Decreased nasal tip rotation	107	36.5	(31.01–42.03)%
Increased nasal tip rotation	13	4.4	(2.08–6.79)%
Broad nasal tip shape	79	27.0	(21.88–32.04)%
Square nasal tip shape	17	5.8	(3.13–8.48)%

### 3.3 Sex-wise prevalence of common nasal deformities

External nasal deviation emerged as a notable deformity, with a higher prevalence among males (65.6%, *n* = 84) compared to females (46.1%, *n* = 76). The statistical analysis confirmed this significant difference, with a chi-square value of 11.13 and a *p*-value of 0.001. In contrast, the broad dorsum deformity exhibited a somewhat comparable distribution between the sexes, with 39.8% (*n* = 51) of males and 31.5% (*n* = 52) of females affected. However, this difference was not statistically significant (chi-square = 2.19, *p* = 0.139).

Regarding the dorsal hump deformity, there was a marginal difference between males (60.2%, *n* = 77) and females (58.2%, *n* = 96). This observation was further supported by a non-significant chi-square value of 0.12 and a *p*-value of 0.733. The saddle nose deformity displayed minimal prevalence across both sexes, with no significant difference observed (chi-square = 0.20, *p* = 0.652).

A noteworthy finding was the prevalence of abnormal nasal tip rotation. Decreased nasal tip rotation was more prevalent among females (40.6%, *n* = 67) than males (31.3%, *n* = 40), while increased nasal tip rotation was more common in females (6.1%, *n* = 10) than males (2.3%, *n* = 3). This disparity was statistically significant, with a chi-square value of 6.06 and a *p*-value of 0.048.

**Figure 3:**
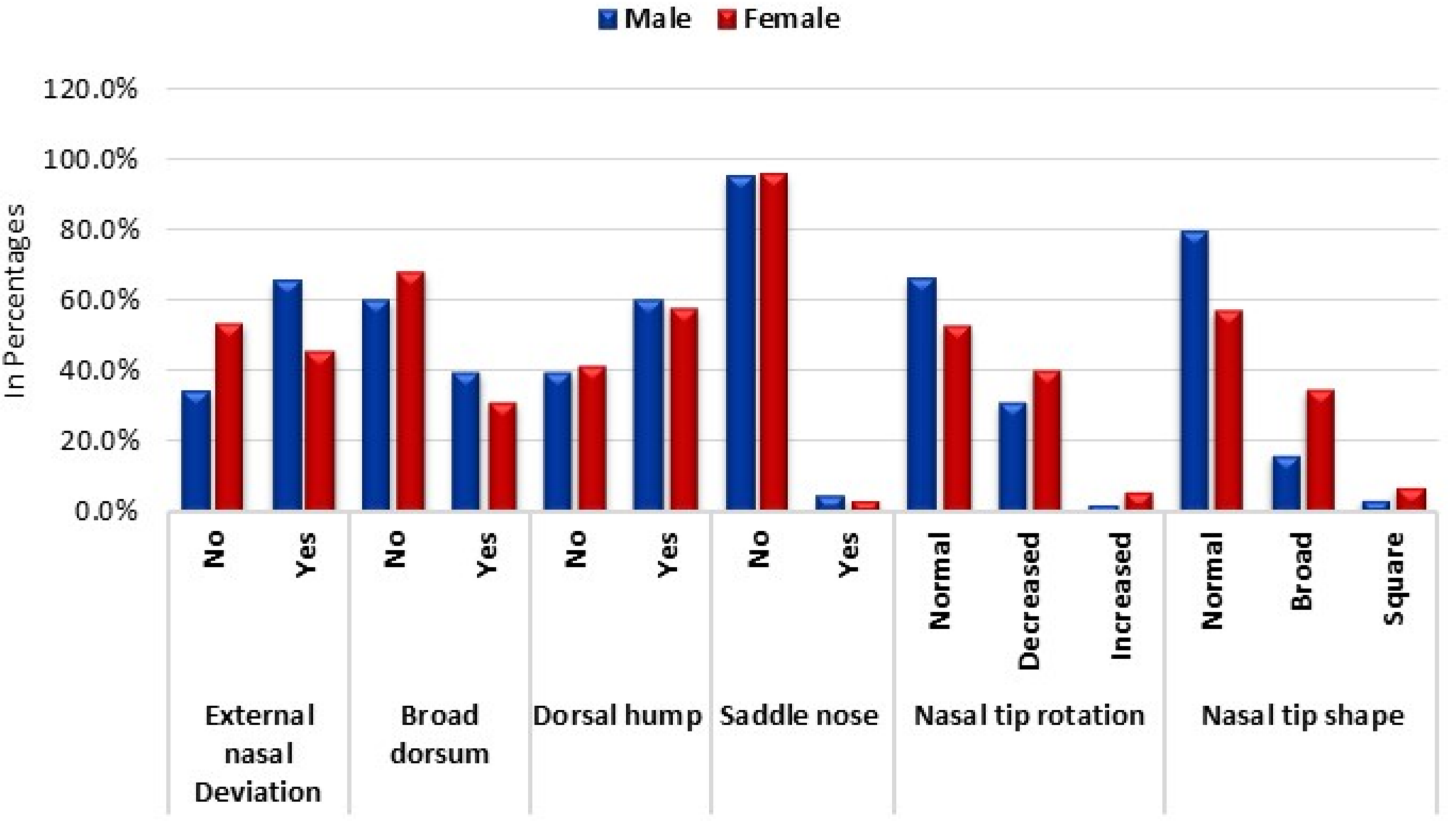
Sex wise prevalence of common nasal deformities.

Lastly, variations in nasal tip shape were also evident. A broad nasal tip shape was more prevalent in females (35.2%, *n* = 58) than in males (16.4%, *n* = 21), while a square nasal tip shape was slightly more common in females (7.3%, *n* = 12) than in males (3.9%, *n* = 5). This difference in nasal tip shape distribution was statistically significant, as indicated by a chi-square value of 16.04 and a *p*-value of less than 0.001 (Table 3; Figure 3).

**Table 3:** Sex wise prevalence of common nasal deformities.

Nasal deformity		Sex			
		Male		Female	
		No.	%	No.	%
External nasal deviation	No	44	34.4%	89	53.9%
	Yes	84	65.6%	76	46.1%
	Significance	chi sq = 11.13, ***p* = 0.001**			
Broad dorsum	No	77	60.2%	113	68.5%
	Yes	51	39.8%	52	31.5%
	Significance	chi sq = 2.19, *p* = 0.139			
Dorsal hump	No	51	39.8%	69	41.8%
	Yes	77	60.2%	96	58.2%
	Significance	chi sq = 0.12, *p* = 0.733			
Saddle nose	No	122	95.3%	159	96.4%
	Yes	6	4.7%	6	3.6%
	Significance	chi sq = 0.20, *p* = 0.652			
Nasal tip rotation	Normal	85	66.4%	88	53.3%
	Decreased	40	31.3%	67	40.6%
	Increased	3	2.3%	10	6.1%
	Significance	chi sq = 6.06, ***p* = 0.048**			
Nasal tip shape	Normal	102	79.7%	95	57.6%
	Broad	21	16.4%	58	35.2%
	Square	5	3.9%	12	7.3%
	Significance	chi sq = 16.04, ***p* < 0.001**			

*p*-value less than 0.05 is deemed to be statistically significant.

### 3.4 Age-wise prevalence of common nasal deformities

The study examined the distribution of various nasal deformities across different age groups, aiming to understand how age might influence the prevalence of these deformities.

**Figure 4:**
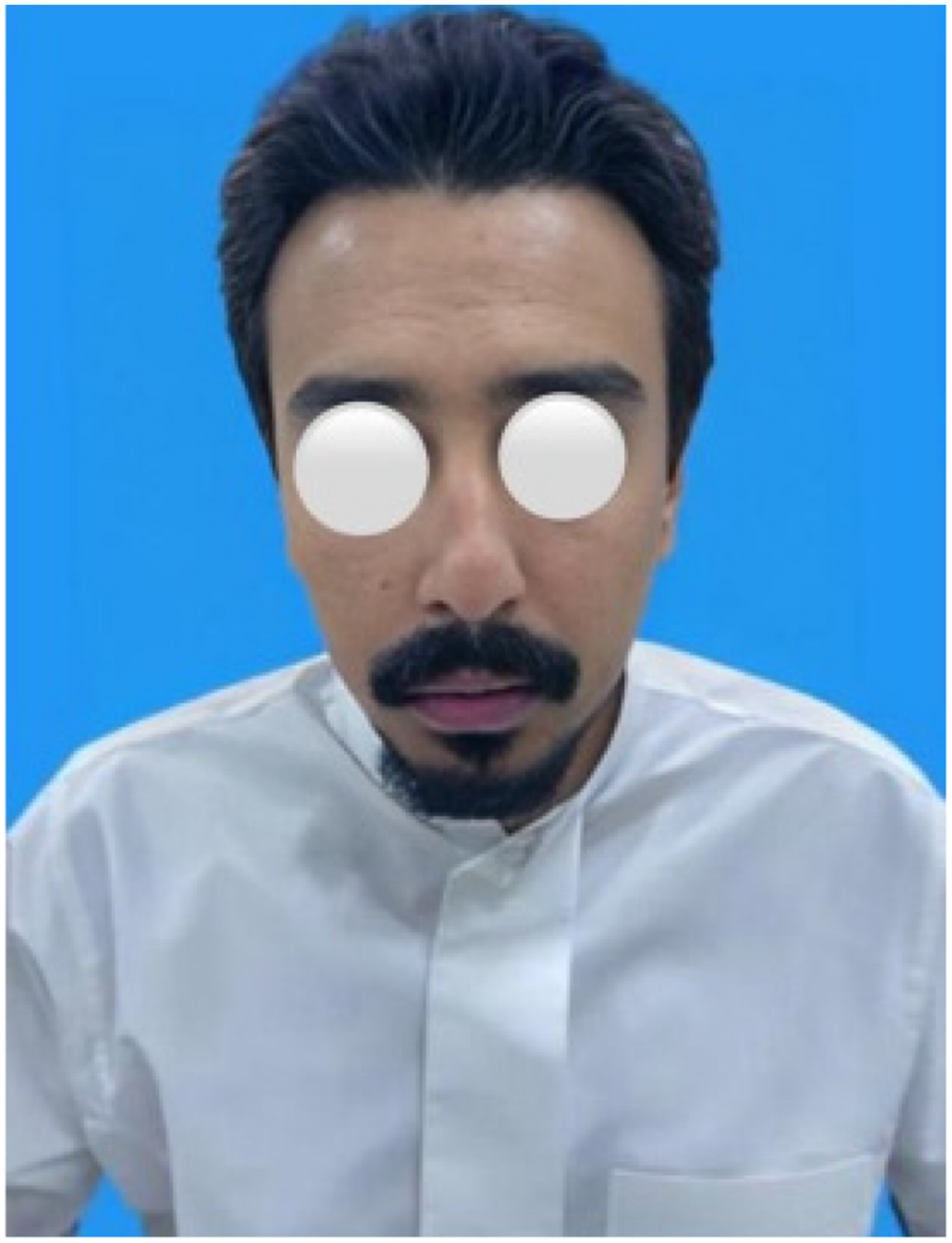
Deviation of the external nose.

For external nasal deviation (Figure 4), the prevalence was relatively consistent across the age groups. In the youngest age bracket (15–24 years), 51.4% (*n* = 36) had the deformity, compared to 53.6% (*n* = 89) in the 25–34 year group, 64.4% (*n* = 29) in the 35–44 year group, and 50.0% (*n* = 6) in the 45–54 year group. However, these variations were not statistically significant (chi-square = 2.21, *p* = 0.530).

Similarly, the distribution of broad dorsum showed no significant difference across age groups. The prevalence ranged from 37.1% to 33.3% across the age groups, with a chi-square value of 0.99 and a *p*-value of 0.805.

**Figure 5:**
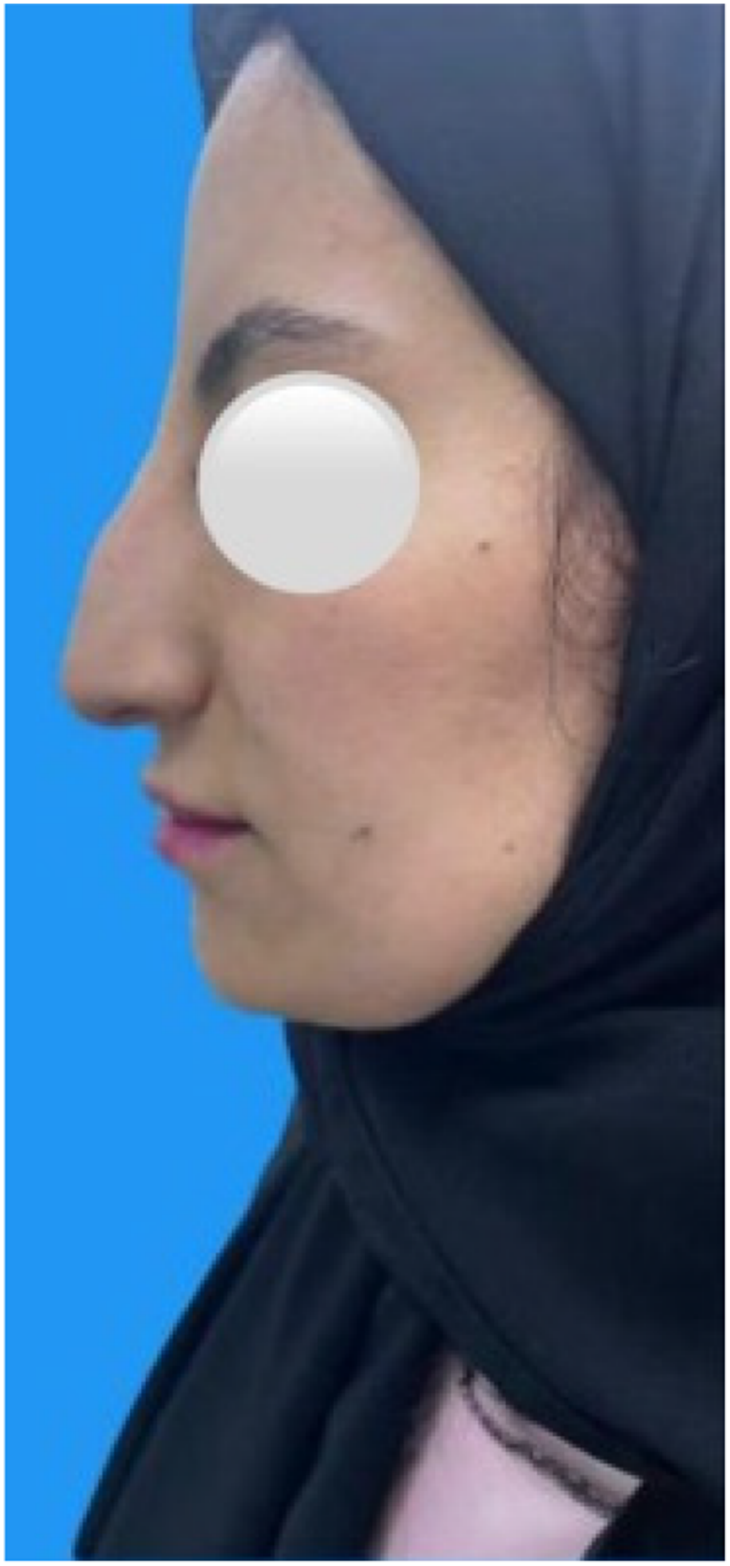
Dorsal nasal hump.

The dorsal hump (Figure 5) deformity displayed a somewhat variable trend, with the highest prevalence observed in the youngest age group (67.1%, *n* = 47) and gradually decreasing in older age groups. Despite this trend, the differences were not statistically significant (chi-square = 5.18, *p* = 0.159).

For saddle nose deformity, the majority in each age group did not have the condition, with prevalence rates above 91%. The chi-square test confirmed that the differences across age groups were not significant (chi-square = 3.42, *p* = 0.331).

In terms of nasal tip rotation, no significant variations were observed across age groups. The prevalence of decreased nasal tip rotation ranged from 33.3% to 41.7%, with a chi-square value of 2.01 and a *p*-value of 0.919.

**Figure 6:**
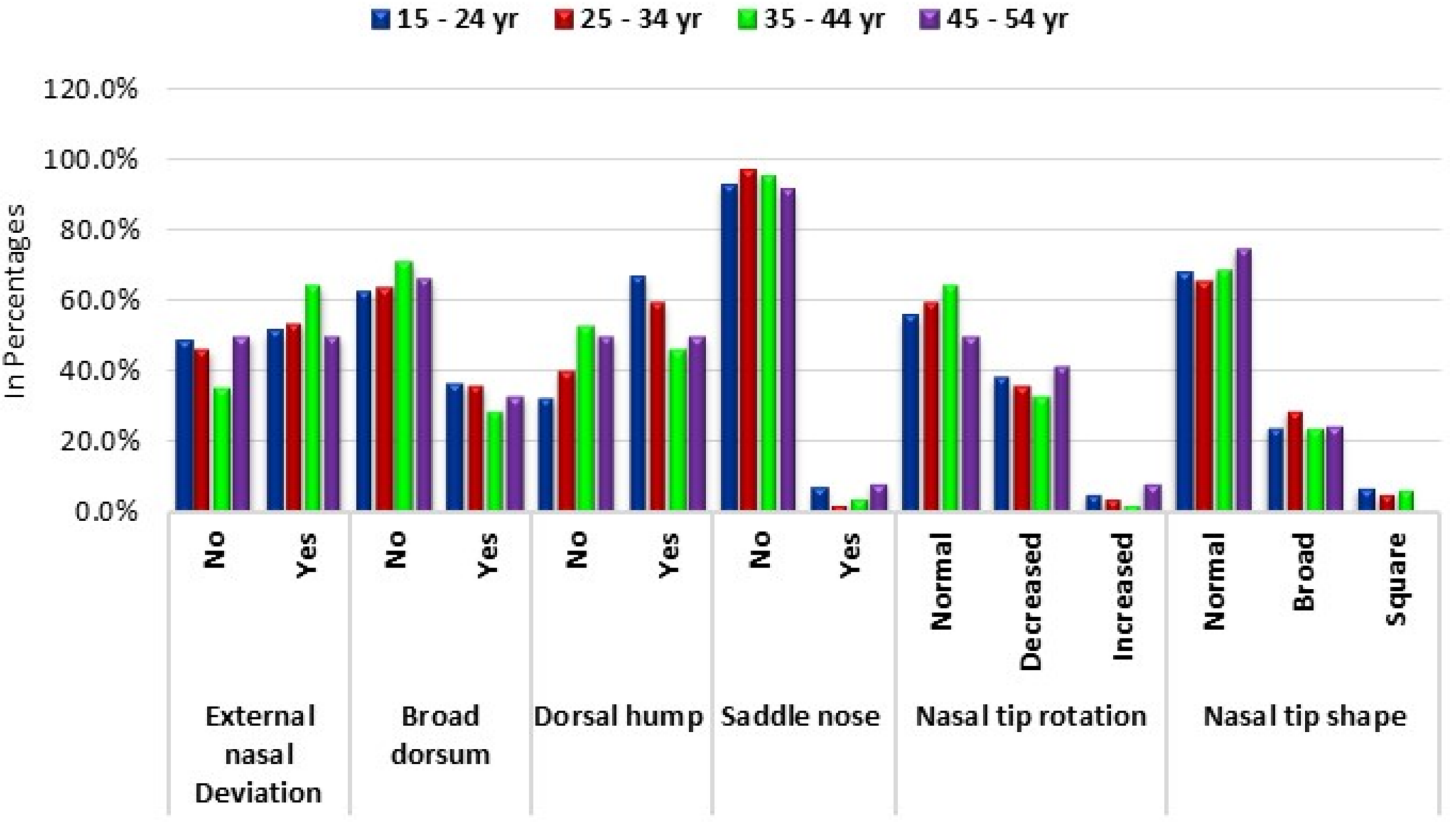
Age wise prevalence of common nasal deformities.

Finally, the distribution of nasal tip shapes remained relatively stable across age groups. The prevalence of a normal nasal tip shape was consistently high, ranging from 65.7% to 75.0%, with no significant differences observed (chi-square = 1.76, *p* = 0.940) (Table 4; Figure 6).

**Table 4:** Age wise prevalence of common nasal deformities.

Nasal deformity		Age							
		15–24 years		25–34 years		35–44 years		45–54 years	
		No.	%	No.	%	No.	%	No.	%
External nasal deviation	No	34	48.6%	77	46.4%	16	35.6%	6	50.0%
	Yes	36	51.4%	89	53.6%	29	64.4%	6	50.0%
	Significance	chi sq = 2.21, *p* = 0.530							
Broad dorsum	No	44	62.9%	106	63.9%	32	71.1%	8	66.7%
	Yes	26	37.1%	60	36.1%	13	28.9%	4	33.3%
	Significance	chi sq = 0.99, *p* = 0.805							
Dorsal hump	No	23	32.9%	67	40.4%	24	53.3%	6	50.0%
	Yes	47	67.1%	99	59.6%	21	46.7%	6	50.0%
	Significance	chi sq = 5.18, *p* = 0.159							
Saddle nose	No	65	92.9%	162	97.6%	43	95.6%	11	91.7%
	Yes	5	7.1%	4	2.4%	2	4.4%	1	8.3%
	Significance	chi sq = 3.42, *p* = 0.331							
Nasal tip rotation	Normal	39	55.7%	99	59.6%	29	64.4%	6	50.0%
	Decreased	27	38.6%	60	36.1%	15	33.3%	5	41.7%
	Increased	4	5.7%	7	4.2%	1	2.2%	1	8.3%
	Significance	chi sq = 2.01, *p* = 0.919							
Nasal tip shape	Normal	48	68.6%	109	65.7%	31	68.9%	9	75.0%
	Broad	17	24.3%	48	28.9%	11	24.4%	3	25.0%
	Square	5	7.1%	9	5.4%	3	6.7%	0	0.0%
	Significance	chi sq = 1.76, *p* = 0.940							

## 4 Discussion

This study, encompassing 293 participants, explores the intricate interplay between demographic factors and prevalent nasal deformities, with a specific focus on gender and age distribution. By meticulously analyzing the bio-demographic composition of the sample, the research aims to unravel potential associations that may influence the occurrence of nasal traits. Through a comprehensive examination of common nasal deformities, such as external nasal deviation, dorsal hump, and variations in nasal tip rotation and shape, this study sheds light on patterns that may contribute to a deeper understanding of nasal anatomy and aesthetics across diverse demographic profiles.

Our study, encompassing 293 participants, discovered a slight female predominance at 56.3%, with males comprising 43.7%. Among the participants, 56.7% fell within the 25–34 age group, while 67.2% held bachelor's degrees, 29.0% had qualifications below that level, and 3.8% possessed advanced degrees. Alsubeeh et al. focused on 2,740 respondents, reporting a higher female majority (84.38%) with a mean age of 20.9 ± 5.54 years (8). In Alshami et al.'s study with 306 student participants, 80.7% were females, most aged 18–25 (97.4%), and the majority were studying medicine (65.7%) (9). Finally, Alsulaiman et al. surveyed 413 female high school students, with the majority aged 17 years (48.9%), residing in Riyadh City (77.5%), and having parents with bachelor's degrees or higher (father, 71.4%; mother, 66.1%). Interestingly, 21.1% noted a connection with someone who underwent rhinoplasty (10).

Our study found that dorsal hump deformity was the most prevalent deformity, affecting 59.0% of participants, followed closely by external nasal deviation at 54.6%. Decreased nasal tip rotation was prevalent in 36.5% of participants, with a saddle nose and increased nasal tip rotation being less frequent. Alsubeeh et al. reported functional complaints in their study, with nasal obstruction (56.9%), nasal stuffiness (51%), and trouble breathing (50.2%) being frequently noted (8). Alshami et al. found that a majority of students were content with their noses (60.1%), with 40.2% expressing no perceived need for rhinoplasty. Interestingly, 30.4% had a family history of cosmetic interventions, and 65.0% felt that rhinoplasty was socially accepted in Jeddah (9). In the Alsulaiman et al. study, students were knowledgeable about rhinoplasty complications, with dissatisfaction with the new nose (70.5%), headache (70.2%), and nose blockage (68%) being recognized as common issues (10). Alharethy et al. studied 248 Saudi patients, revealing common external nasal deformities such as broad nasal dorsum (65.7%), bulbous nasal tip (62.1%), and nasal deviation (60.5%) (11).

Our study observed that external nasal deviation was significantly more prevalent in males (65.6%) than females (46.1%), as confirmed by a chi-square value of 11.13 (*p* = 0.001). Additionally, decreased nasal tip rotation was significantly more common in females (40.6%) than males (31.3%) (*p* = 6.06, *p* = 0.048), and variations in nasal tip shape were statistically significant, with broad nasal tip shape more prevalent in females (35.2%) and square nasal tip shape slightly more common in females (7.3%) (*p* = 16.04, *p* < 0.001). Alsubeeh et al. reported a prevalence of 44.7% for individuals considering revision rhinoplasty, primarily driven by the desire for further aesthetic improvement in an already acceptable result. The most commonly subjectively reported cosmetic complaint was a poorly defined nasal tip (32.35%) (8). In the Alharethy et al. study, broad nasal dorsum (65.7%), bulbous nasal tip (62.1%), and nasal deviation (60.5%) were the most common external nasal deformities. Deviation was more frequent in the 20–24 age group (44.0%), and an under-rotated nasal tip was most common in the 20–24 age group (*P* = 0.047). Increased columellar show was more common in the oldest age group (*P* = 0.04) (11).

Our study revealed that the prevalence of external nasal deviation and broad dorsum did not significantly differ across age groups, with chi-square values of 2.21 (*p* = 0.530) and 0.99 (*p* = 0.805), respectively. Although dorsal hump deformity showed a variable trend with age, the differences were not statistically significant (chi-square = 5.18, *p* = 0.159). Saddle nose deformity, nasal tip rotation, and nasal tip shapes also exhibited no significant variations across age groups, as indicated by chi-square values of 3.42 (*p* = 0.331), 2.01 (*p* = 0.919), and 1.76 (*p* = 0.940), respectively. In the Alharethy et al. study, which included 248 patients, the most common external nasal deformities were a broad dorsum (65.7%), a bulbous columella (62.1%), and a deviation (60.5%). The prevalence of upward columella was noted to be higher in older patients, while deviation decreased with age. While there are some similarities in the prevalence of specific deformities, the age-related trends in nasal deformities differ between the two studies, highlighting the complexity and variability in the presentation of nasal features across populations (11).

The exploration of gender and age distribution within our cohort revealed significant associations with specific nasal traits, emphasizing the complex interplay between anatomy and demographic variables. Notably, external nasal deviation exhibited gender disparities, with males showing a higher prevalence. Additionally, the age-related trends in nasal deformities highlighted the nuanced presentation of nasal features across different age groups. Functional complaints and attitudes towards rhinoplasty further added depth to the understanding of the multifaceted nature of nasal anatomy and aesthetics. These collective insights underscore the importance of considering demographic factors in the assessment of nasal features, contributing to a broader and more nuanced understanding of nasal deformities in diverse populations.

## Data Availability

The original contributions presented in the study are included in the article/Supplementary Material, further inquiries can be directed to the corresponding author.
